# Hospital care for elderly COVID-19 patients*

**DOI:** 10.1590/1518-8345.4649.3396

**Published:** 2020-11-06

**Authors:** Jack Roberto Silva Fhon, Luipa Michele Silva, Zoila Esperanza Leitón-Espinoza, Fernanda de Brito Matiello, Jessica Silva de Araujo, Rosalina Aparecida Partezani Rodrigues

**Affiliations:** 1 Universidade de São Paulo, Escola de Enfermagem, São Paulo, SP, Brazil.; 2 Universidade Federal de Goiás Regional Catalão, Escola de Enfermagem, Catalão, GO, Brazil.; 3 Universidad Nacional de Trujillo, Escuela de Enfermería, Trujillo, La Libertad, Peru.; 4 Universidade de São Paulo, Escola de Enfermagem de Ribeirão Preto, PAHO/WHO Collaborating Centre for Nursing Research Development, Ribeirão Preto, SP, Brazil.

**Keywords:** Aged, Coronavirus Infections, Admitting Department, Hospital, Newspaper Article, Health Personnel, Pandemics, Idoso, Infecções por Coronavírus, Serviço Hospitalar de Admissão de Pacientes, Artigo de Jornal, Pessoal de Saúde, Pandemias, Anciano, Infecciones por Coronavirus, Servicio de Admisión en Hospital, Artículo de Periódico, Personal de Salud, Pandemias

## Abstract

**Objective:**

to analyze the newspaper articles on hospital care for elderly COVID-19 patients in online newspapers.

**Method:**

documentary, retrospective, descriptive and exploratory research. The data were collected from articles published on open-access websites of 12 newspapers from the following countries: Brazil, Spain, United States, France, Italy and Portugal.

**Results:**

out of 4,220 newspaper articles identified in this regard, 101 were selected after applying the inclusion criteria, the majority coming from Italy. The data analysis revealed three thematic categories: the care for patients with COVID-19 in the health system; the work process of the health team and its concern with contagion; and ethical dilemma in care for the elderly during hospitalization.

**Conclusion:**

the COVID-19 pandemic presented itself quickly and was widely reported in all countries. The health systems need to reorganize for care to the global population, especially the elderly, considering their weaknesses and also the lack of prior professional training to offer care to this population.

## Abstract (pt)

**Objetivo:**

analisar as matérias jornalísticas sobre o atendimento hospitalar aos idosos com COVID-19 nos veículos de comunicação *online*.

**Método:**

pesquisa documental, retrospectiva, descritiva e exploratória. Os dados foram coletados de matérias publicadas em *websites* de acesso livre de 12 jornais dos seguintes países: Brasil, Espanha, Estados Unidos, França, Itália e Portugal.

**Resultados:**

do total de 4.220 matérias jornalísticas identificadas a esse respeito, 101 foram selecionadas após aplicação dos critérios de inclusão, a maioria proveniente da Itália. A análise dos dados revelou três categorias temáticas: O atendimento ao paciente com COVID-19 no sistema de saúde; Processo de trabalho da equipe de saúde e sua preocupação com o contágio; e Dilema ético no atendimento ao idoso durante a internação hospitalar.

**Conclusão:**

a pandemia da COVID-19 se apresentou de forma rápida e foi bastante noticiada em todos os países. É necessário que os sistemas de saúde se reorganizem para o atendimento à população mundial, sobretudo ao idoso, considerando suas fragilidades e também a ausência de capacitação profissional prévia para oferecer assistência a essa população.

## Abstract (es)

**Objetivo:**

analizar las noticias periodísticas sobre la atención hospitalaria a los adultos mayores con COVID-19 en los medios de comunicación *on-line*.

**Método:**

estudio documental, retrospectivo, descriptivo y exploratorio. Los datos fueron recolectados de materias publicadas en las páginas *web* de libre acceso de 12 periódicos de Brasil, España, Estados Unidos, Francia, Italia y Portugal.

**Resultados:**

de 4,220 noticias periodísticas identificadas sobre el tema de estudio, 101 fueron seleccionados posterior a los criterios de inclusión, siendo la mayoría de Italia. Al análisis de los datos se identificó tres categorías temáticas: *La atención al paciente con COVID-19 en el sistema de salud; Proceso del trabajo del equipo de salud y su preocupación al contagio* y *Dilema ético en la atención al adulto mayor durante su hospitalización*.

**Conclusión:**

la pandemia de COVID-19 se presentó de forma rápida y fue difundida en todos los países. Es necesario que los sistemas de salud se reorganicen para la atención de la población en el mundo, especialmente al adulto mayor, tomando en cuenta su fragilidad y también la ausencia de una previa capacitación profesional para ofrecer atención a esta población.

## Introduction

Public health has been facing one of the biggest pandemics of this century, caused by the novel coronavirus (SARS-CoV-2), which causes COVID-19. The first cases of the disease were reported in Wuhan-China in December 2019^(^ 1 ^)^. COVID-19 is an acute respiratory disease, transmitted from person to person, which presents high mortality among the elderly^(^ 2 ^)^. The mortality rate in this population is around 14.8%^(^ 3 ^)^ and, in people with underlying medical conditions such as cardiovascular diseases (13.2%), with diabetes mellitus (9.2%), arterial hypertension (8.4%), chronic respiratory diseases (8.0%) and cancer (7.6%)^(^ 4 ^)^.

Being a respiratory disease, the incubation period varies between five and 14 days, and the transmission period is five days after the appearance of the first symptoms^(^ 5 ^)^. The differential of this disease is the severe acute respiratory syndrome (SARS), which has affected between 17% and 29% of patients. In addition, in 75% of these, atypical bilateral pneumonia occurs, detected by computerized tomography^(^ 6 ^)^.

Given the severity of the disease, on January 1, 2020, the World Health Organization (WHO) started several actions to combat the outbreak. COVID-19 was already considered a public health emergency on January 30 and, on March 11, it became characterized as a pandemic, after infecting 118,000 people in 114 countries and leading to 4,291 deaths^(^ 7 ^)^. This disease is highly infectious and 20% of the infected people develop respiratory disorders^(^ 6 ^)^.

On May 25, 2020, WHO and the Johns Hopkins Center for Health Security confirmed 5,453,784 cases of the disease and 345,886 deaths in 185 out of 195 countries in the world^(^ 8 ^)^. In addition to the effects on public health, which show the deterioration of public health systems in the face of the demand for care, the crisis caused by the pandemic causes serious problems in the economy and accentuates the social inequality of the population, in view of the unavailability of equipment and protection products for all, in an equal manner^(^ 9 ^)^.

Furthermore, a collapse of the health systems is noted in different countries without infrastructure, human resources, equipment and material for simultaneous care to thousands of infected patients. In addition, the hospitalization period in the Intensive Care Unit (ICU) has been long, which increases the waiting time for patients in severe conditions. This situation requires care protocols and imposes on health professionals the difficult decision to choose who can live or die^(^ 10 ^)^. The recommendation not to offer ventilation equipment to people over 80 years of age^(^ 11 ^)^ when the demand exceeds the supply aggravates this situation even further.

Currently, although hospitals are caring for and treating people infected with this disease using more advanced resources, there is worldwide carelessness for elder elderly with suspected or confirmed COVID-19. Thus, given the above and considering that the media influences public opinion on several topics, we aim to understand how the worldwide written press has reported on the hospital treatment offered to elderly people with COVID-19. Therefore, the following guiding question was elaborated: How are newspaper articles on hospital care for elderly patients with COVID-19 reported in online newspapers?

As online dissemination represents a revolution in the continuous news production, distribution, and updating model, expanding the knowledge of content published on websites about health, as well as about its interrelations and determinants, is important to inform and educate society. In addition, it can influence individual actions, the general population, the medical community, and public policy makers^(^ 12 ^)^.

In that sense, the objective of this article is to analyze the newspaper articles on hospital care for elderly COVID-19 patients in online newspapers.

## Method

Documentary, retrospective, descriptive and exploratory research. Newspaper articles published on websites of 12 open-access newspapers from six countries were analyzed: Brazil, Spain, the United States, France, Italy, and Portugal.

The sample consisted of newspaper articles that met the following inclusion criteria: disseminate news about COVID-19 between January 1 and April 20, 2020; provide open and full access to newspaper articles about care for the elderly with COVID-19 in the hospital context and use the following terms: COVID-19 (coronavirus), elderly (*viejos, abuelos, personas mayores,* elderly, old, older people*, vecchio*), ICU (*UCI*) and doctor (*médico*). Both the choice of these countries for the analysis and the selection of the articles took into account two main factors: number of cases of the pandemic and language mastered by the authors of the study. It is important to clarify that newspaper articles published in various formats were analyzed, including news items, reports, articles, interviews, editorials, among others.

Data collection included the stages of identification, selection and eligibility. In the first phase, we identified a total of 12 newspapers with open access and that published full articles. In the second stage, the words used in the search were: elderly, coronavirus, COVID-19 and hospital, standardized according to the languages of each country. In the third stage, after reading the identified material, the articles that met the pre-established criteria were selected, totaling 101 articles.

A database was built to standardize the terms used by the different newspapers and thus facilitate the comparison during the analysis. Nevertheless, care was taken to maintain the context and to respect, in each language, the various forms of writing. All stages of the research and in production of the article met the criteria of the Consolidated Criteria for Reporting Qualitative Research^(^ 13 ^)^.

To analyze the selected subjects, the thematic analysis technique was adopted, which is used in health to analyze the ideas expressed, words or other symbols that make up the content of the articles^(^ 14 ^)^.

All newspaper articles were grouped according to the country of origin and composed a database, which was subsequently analyzed using the software *Interface de R pour les Analyses Multidimensionnelles De Textes et de Questionnaires* - IRAMuTeQ 0.6 alpha 3, Brazilian version. This software develops statistical analyses on text segments, individual pictures and words^(^ 15 ^)^.

The first analysis permitted the construction of word clouds, which represent a simpler lexical analysis, which groups and graphically organizes the words according to how frequently they are used. This analysis was chosen to identify the contents most reported in each newspaper and considered the frequency greater than ten, in order to generate more understandable figures.

In the second analysis, the thematic categories were constructed, which emerged from reading and textual analysis using the Reinert method, known as descending hierarchical classification (DHC), which permits analyzing the occurrence of terms in a specific segment of the text. In this type of analysis, the software permits identifying co-occurrences of terms in the same segments, distributing them in classes by proximity to rank the relative presence of each term in the created classes. One DHC was elaborated for each language, as the *software* does not permit the joint analysis of different languages. Based on the DHC, the phrases with thematic proximity were extracted and then grouped into the categories described by the researchers^(^ 16 ^)^.

Two researchers validated the analyses to guarantee results in line with the objectives of the study and the proposed theme. Then, two authors validated the content in order to respect the criteria of scientific publication and ensure the appropriate language.

As the study used only publicly accessible and free information available on the websites of selected newspapers, no ethical approval was necessary, in accordance with CNS Resolution 510/2016^(^ 17 ^)^.

## Results

Figure 1 shows the results obtained from the search in newspapers circulating online. According to the collected data, in the 12 selected newspapers, 4,220 articles were identified on the research subject, but only 101 articles met the inclusion criterion.

**Figure 1:** Distribution of the newspaper articles analyzed according to reading order, countries included and keywords mentioned. Brazil, January/April 2020

Country	Newspapers	Words	Number of articles	Number of articles selected
Brazil	O Globo O Estado de São Paulo	*Idoso, Velho, COVID-19, Coronavírus, Hospital*	451 226	05 03
Spain	El País ABC	*Anciano, Mayores, Viejo, COVID-19, Coronavírus, Hospital*	154 229	09 04
United States	*Washington Post* *Daily News*	Elderly, Older, Elder, Old, COVID-19, Coronavírus, Hospital	115 864	07 08
France	*20 Minutes* *Le Nouvel Obs*	Âgées, *COVID-19, Coronavirus, Hôpital*	1,000 155	16 03
Italy	*La Repubblica* *Il Giorno*	*Anziani, Coronavírus, Ospedale*	354 299	24 09
Portugal	*Público* *Observador*	*Idoso, Velho, COVID-19, Coronavírus, Hospital*	126 247 373	06 07
Total				101

The French newspaper *20 Minutes* concentrated the largest number of publications with the keywords chosen (1,000) and *The Washington Post*, from the United States, the smallest (115). Italy obtained the highest number of selected newspaper articles (33), followed by France (19) and the United States (15).

**Figure 2:**
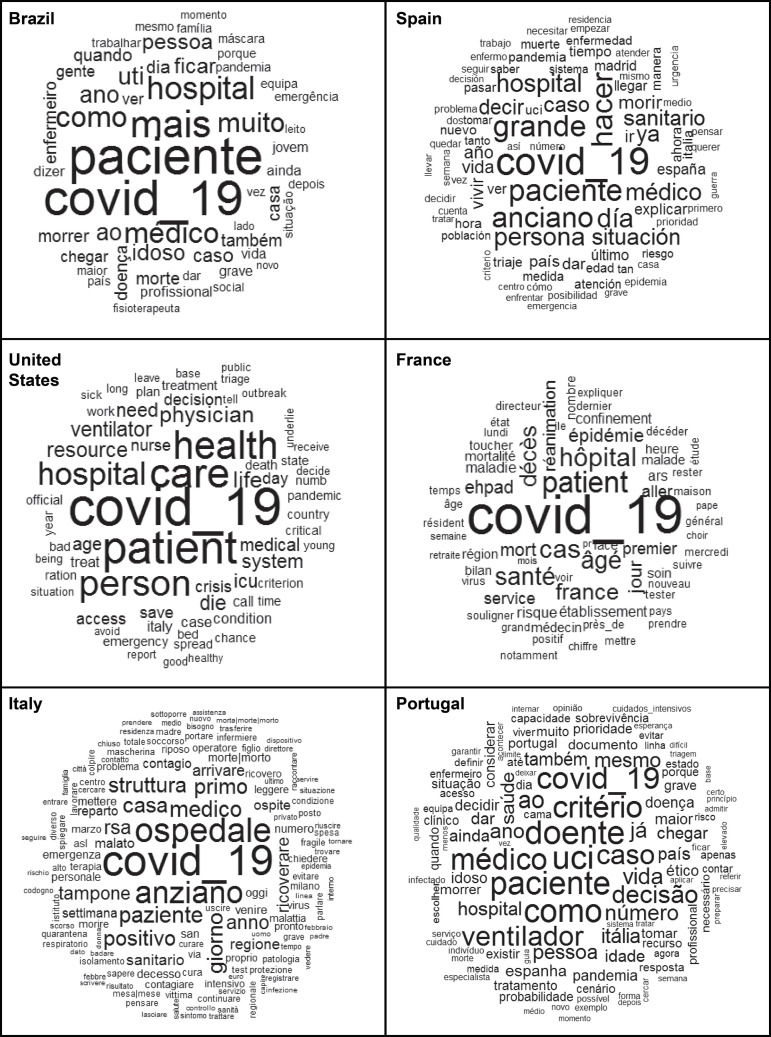
Word clouds extracted from newspaper articles (n=101) on hospital care for elderly with COVID-19, Brazil, January/April 2020

As for the content of the newspaper articles, the most frequent terms in the word cloud were: in Brazil, *paciente* (patient - f = 84); COVID-19 (f = 80); *mais* (more - f = 59); *hospital* (f = 48) and *médico* (physician - f = 45); in Spain, COVID-19 (f = 67); *paciente* (patient - f = 51); *grande* (large - f = 50); *hacer* (to do - f = 50) and *anciano* (elderly - f = 49); in the United States, COVID-19 (f = 97); patient (f = 80); care (f = 72); health (f = 69) and person (f = 62); in France, COVID-19 (f = 110); *patient* (patient - f = 54); *hôpital* (hospital - f = 47); *cas* (cases - f = 47) and âgé (elderly - f = 43); in Italy, COVID-19 (f = 149); *ospedale* (hospital - f = 113); *anziano* (elderly - f = 103); *paziente* (patient - f = 73) and *medico* (physician - f = 72); and in Portugal, *UCI* (Intensive Care Unit - f = 70); *doente* (ill - f = 69); *paciente* (patient - f = 68); COVID-19 (f = 64) and *critério* (criterion - f = 64). These data are detailed below (Figure 2).

Most of the newspaper articles came from Italy and the thematic analysis of the data permitted the construction of three categories: *Care for the patient with COVID-19 in the health system; The work process of the health team and its concern with the contagion*; and *Ethical dilemma in care for the elderly during hospitalization*.

In the category care for patients with COVID-19 in the health system, the contents of the publications showed the difficulties each country faced in view of the pandemic and the need for health systems to support the health professionals, related to safety and other equipment for care to patients hospitalized due to COVID-19, including mechanical ventilators in intensive care unit (ICU) environments. The newspapers highlighted the responsibility of professionals who act on the front line against the disease:

*Las altas tasas de contagio y los graves efectos causados sobre un porcentaje relativamente elevado de nuestra población han llevado al límite a nuestro sistema sanitario.* (Spain, 9); *Hospitais preparam-se para o impreparável: o aumento de doentes em UCI. Número de doentes críticos com COVID-19 internados em UCI cresceu 20 vezes em menos de três semanas* (Portugal, 6).

In the category *Work process of the health team and its concern with contagion*, the contents of the newspaper articles pointed to the daily concern of the press with the work process of health professionals and with their own safety, due to the risk of contagion with COVID-19, which increases as more patients are cared for at the ICU. In this sense, in the different countries, newspaper articles can be found that highlight security as something essential for all:

*Un employé de l’hôpital gériatrique de Francheville, vers Lyon, est actuellement en réanimation. La série de contaminations est partie d’un salarié de l’hôpital*. (France, 7); *Il sindacato propone varie misure di sicurezza, tra cui «un certificato nel quale specificare la quantità di trattamenti erogati dal singolo operatore sanitario e la quantità di dispositivi di protezione individuale consegnati per ogni giorno di trattamento.* (Italy, 15); À saída do turno, o ritual de retirar o equipamento ainda é mais exigente, porque por cada peça retirada é preciso desinfectar as mãos. Já devidamente equipados e desinfectados, os elementos da equipa, maioritariamente enfermeiros, entram na unidade onde começa um árduo trabalho para salvar os doentes. A Lusa pôde assistir à colocação de um doente em posição de decúbito ventral para melhorar a sua oxigenação que obrigou a um verdadeiro trabalho de equipa. (*Portugal, 7).*

In the category *Ethical dilemma in care for the elderly during hospitalization*, the subjects report that the care of elderly patients in the ICU diagnosed with COVID-19 around the world has increased considerably in health services. The newspapers reported the professionals’ concern with the measures proposed in each country in the face of the pandemic, because they consider that they can harm the care of the elderly, who are the most vulnerable and in need of longer care. They also reported that the decision not to prioritize care for these people in the face of the high demand imposed an ethical conflict on these professionals:

*Dilema ético, os idosos e a metáfora da guerra. Parte da sociedade é tratada como inútil e improdutiva. A metáfora de guerra tem sido utilizada para espelhar a luta que está sendo travada contra a COVID-19.* (Brazil, 6); *Se les dejará morir. El departamento ha elaborado un documento que determinará qué pacientes reciben tratamiento en UCI y cuáles no… un especialista en reanimación y un médico en medicina interna son los encargados de decidir qué paciente ingresará a la UCI. La edad y las enfermedades previas son factores importantes en este sistema de triaje. Pero también lo es tener una familia.* (Spain, 2); *Medical ethicists also have suggested that we should ration ventilators by denying them to patients over a certain age. They argue that treating only the young will be efficient, saving the greatest total life-years.* (United States, 10).

## Discussion

The results obtained using the word cloud technique supported the construction of the thematic categories and show the reflection of the pandemic, its implications for care for the elderly and the measures proposed in different countries for the control of the novel coronavirus. In all analyses, COVID-19 was identified as the main and recurring theme. Nevertheless, the selected contents contain words that refer to elderly patients, indicating the press’ concern with disseminating news about the care for this part of the population.

Some differences between the countries in the dissemination of news about hospital care for elderly people with COVID-19 are noticeable in Figure 2 and in the thematic categories identified, especially in European countries, which experienced the severity of the disease before America, facing the overcrowding of hospitals even before the first cases of deaths were recorded in Brazil. This means that the newspaper articles published in this regard in Brazil in the research period did not yet fully cover the research problem, as occurred in the other countries included in this study.

In Category 1, the importance of providing human and material resources for care to individuals with acute respiratory failure is highlighted, as the major problem reported in the media surveyed is the ability of health systems to deal with demand fluctuations, especially with the increase of elderly patients, who need respiratory support.

The difficulties to offer hospital care to more severe patients stand out, due to the very restriction of ICU hours, the limited facilities, and the insufficient number of professionals prepared for this care. In addition, it should be mentioned that the professionals working in an ICU environment usually present physical exhaustion, receive low wages and deal with inappropriate working conditions^(^ 18 ^)^.

Category two emphasizes the work process of the health team and its concern with contagion. It is important to highlight that, in Brazil, there is a need for professionals to work on the front line. Not infrequently, they also have limited knowledge for care to elderly patients with comorbidities, especially in the course of COVID-19^(^ 19 ^)^, and lack a specific treatment, due to not knowing the pathophysiology of this disease.

Concern about contagion is due to several factors, including the severity of the disease. In Brazil, researchers who analyzed the severity of the pandemic warn that people have been directed to seek primary health care when they identify the first symptoms, unlike other countries, which have created specific services for reception, clinical evaluation, and referral of more severe cases to hospitals^(^ 20 ^)^.

The ethical issues presented in category three are due to the increase in the number of patients who need ICU beds in countries such as Spain, Italy and the United States, which has imposed some ethical dilemmas on health professionals, especially with regard to the choice of who may or may not use a mechanical respirator. This situation can be observed in Figure 2, with its specific words, and identified in Category 3 of the newspapers analyzed. As the emerging epidemic is leading to a substantial increase in the number of patients requiring prolonged ventilatory support for acute respiratory failure, serious imbalances have occurred between the clinical needs of the population and the general availability of ICU resources^(^ 10 ^)^.

The ethical dilemmas were identified in the articles published by different newspapers, in which governments indicated guidelines for the care of patients with COVID-19. In Italy, where the ethical issue became more evident, the recommendations included the allocation of resources to the ICU; screening related to the age limit, the presence of comorbidities and the functional *status* of any critical patient upon entering the ICU; the guidelines for early medical care to patients with severe chronic diseases; and the application of palliative care after suspension of treatment in the ICU, when severe complications arise^(^ 10 ^)^.

In the United States, there was an attempt to maximize benefits, treating all patients equally, promoting and rewarding the instrumental value, and granting priority to the poorest. The specific recommendations to allocate medical resources during the COVID-19 pandemic were: maximize benefits; prioritize health professionals; do not allocate patients in order of arrival so that, for those with similar prognoses, equality should be invoked and operationalized through random allocation, like a lottery; be sensitive to evidence; recognize participation in research, and; apply the same principles to all patients with COVID-19 and non-COVID-19^(^ 21 ^)^.

All newspapers published news from their own countries and others that were being affected by the coronavirus. And in the understanding of the categories, it should be kept in mind that there were differences in the spread of the disease within each country, the care of the elderly by the health system, and how the authorities dealt with this problem.

The first European country to be the focus of the pandemic was Italy, where, until May 10, more than 30,000 deaths by COVID-19 were recorded. The lethality in the country reached 7%, almost twice the global average (3.4%). In addition, 60% of the confirmed cases involved people over 65 years of age^(^ 17 ^)^. The higher incidence in this age range is explained by the country’s population characteristic, in which the elderly represent about 22% of the population^(^ 22 ^)^.

Spain was the third most affected country in Europe, with more than 200 thousand cases^(^ 8 ^)^, 57,106 of which needed hospitalization. In this group, 24.1% were between 70 and 79 years old, 19% between 80 and 89 and 5.1% over 90 years of age^(^ 23 ^)^. In long-term institutions, many elderly people were found dead because the funeral system, burdened by the health crisis, did not support the demand for its services^(^ 24 ^)^.

The fourth most affected country in the European community was France: 176,782 cases and 26,313 deaths^(^ 8 ^)^. On March 15, 2020, people over the age of 75 accounted for 20% of confirmed cases, but 79% of deaths by COVID-19. Individuals between 64 and 74 years represented 14% of confirmed cases and 13% of deaths^(^ 25 ^)^, with a great impact in long-term institutions^(^ 26 ^)^.

Portugal was the last country in Europe to have to deal with COVID-19, which provided a timely opportunity for local authorities to organize themselves and start control and prevention measures earlier^(^ 27 ^)^. In the age composition of the contaminated population, there was a high percentage (20.7%) of people over 65 years of age^(^ 22 ^)^, an aspect that made the implementation of preventive measures even more urgent. Nevertheless, the country, even in the face of several attempts to control the spread, faced a total of 1.6 thousand confirmed cases, especially in the elderly^(^ 28 ^)^.

The United States, another heavily affected country, adopted different measures to control the pandemic over the months, which did not prevent the rapid spread of the virus either, reaching severe records of contamination and deaths, which exceeded 70,000 thousand all over the country^(^ 29 ^-^ 31 ^)^. US data also show that 31% of COVID-19 cases occurred in people over 65 years of age and 6% in individuals over 85 years of age. Patients aged 65 years or older accounted for 45% of hospitalizations, 53% of ICU admissions and 80% of deaths though. In addition, the lethality rate increased with age: from 3-5% between 65-74 years, 4-11% between 75-84 years to 10-27% over 85 years of age^(^ 32 ^)^.

In Brazil, the number of infected patients increased considerably, reaching 363,211 and 22,666 deaths on May 25^(^ 8 ^)^, with a lethality rate of 6.8%^(^ 33 ^)^. The first cases reported as fatal by COVID-19 were elderly, who, according to studies, have a higher mortality rate, especially when hospitalized (rates between 11 and 15%). Approximately 10% of the infected elderly develop the severe form of the disease and 5% should receive treatment in intensive care units^(^ 6 ^,^ 34 ^)^. This information supports the data in Figure 2, as it shows differences in the countries studied, that is, that the elderly are the population with the greatest vulnerability to the disease and that, when hospitalized, the risk of death increases.

In Brazil, most of the elderly with COVID-19 are being treated in hospitals and the peak of the pandemic has not yet been established, which generated little news in this regard in the course of the study period. According to research, the disease was introduced into the country by people who had been in other countries and returned from travel. At first, these individuals were isolated, as well as their contacts, in order to prevent the spread of the virus^(^ 35 ^)^.

Most deaths among the elderly are related to chronic diseases, such as cardiovascular diseases. This entails important implications for the way in which public health and clinical responses should be developed, but this problem has been ignored in high^(^ 36 ^)^, middle and low-income countries, which house 69% of the world’s population aged ≥60 years and where health systems are weaker and are therefore collapsing more rapidly with the increase in the number of patients^(^ 19 ^)^.

In Spain, Italy, France and the United States, the growing number of people with COVID-19 cared for in nursing homes or similar institutions was worrying. Most of the residents in these institutions, for the most part, are highly dependent on the caregiver or health professional, and an outbreak in these places can affect up to 60% of the population^(^ 37 ^)^, with severe implications for the well-being and, potentially, the survival of their residents^(^ 19 ^)^. That is probably why the newspapers have published more articles in this respect and the professionals were contaminated because they were dealing with a fragile population highly affected by the virus.

According to the data shown in Figure 1, measures to combat the disease were taken in order to avoid the overcrowding of hospitals and, therefore, the collapse of the health system. Countries like Spain, France, Italy, Portugal and the United States have established the quarantine, that is, restriction of movement and isolation of symptomatic individuals and healthy people who may have been exposed to the virus, with the aim of monitoring their symptoms and ensuring the early detection of cases^(^ 38 ^)^. It is important to mention that, even in these situations, according to Article 3 of the International Health Regulation^(^ 39 ^)^, dignity, human rights and fundamental freedoms of persons have to be fully respected.

The adoption of quarantine measures at the onset of the pandemic can postpone the spread of the disease in a country or area, postpone the peak in regions where local transmission is already occurring, or both. If not implemented properly, however, it can also create additional sources of contamination and spread of the disease^(^ 38 ^)^.

In Brazil, the Ministry of Health implemented control and prevention measures determined by the federal, state, and municipal governments, the most widespread being social isolation. This measure has caused controversy, as many authorities are skeptical about the effectiveness of these actions or fear severe losses in other areas, such as the economy. The population has tried to follow the recommendations, however, in order to prevent the progression of this disease^(^ 40 ^)^.

It is interesting to note that, amidst a pandemic, all countries seek to follow WHO recommendations to prevent a large number of deaths and the collapse of the health care network. Research has been carried out to ascertain which actions were effective for flattening the curve. China, Japan, Thailand, and South Korea adopted the use of the mask, but it was in the Czech Republic, where the use was mandatory, that the number of new cases of COVID-19 increased much slower when compared to other European countries^(^ 41 ^)^. In Brazil, some states are establishing the mandatory use of masks and, even so, already face an overload of health services. This information is not published in scientific journals, but the increase in new cases is reported daily, and urgent protective measures are needed.

Among the elderly, high rates of infectivity and mortality have already been proven, to the extent that, in total, the newspapers have reported more than 100 news items about the care of the elderly in hospitals and deaths resulting from this severity. According to researchers, the high infectivity of SARS-CoV-2 cannot yet be faced using vaccines, which exponentially raises the risks and explains the need for non-pharmacological interventions such as the use of masks, social distancing and others, in order to contain the spread of the virus^(^ 42 ^)^.

In this pandemic, never before has the right to life become so important, as it is inherent in the human rights of all people, without discrimination based on age. This right is largely protected in various documents: Universal Declaration of Human Rights; American Convention on Human Rights; International Covenant on Economic, Social and Cultural Rights; International Covenant on Civil and Political Rights; and Inter-American Convention for the Protection of the Human Rights of Older Persons, among others, of which most countries participate. In this pandemic context, it is important to also recall the initiative of the *United Nations*, in 2011, at the Second World Assembly on Aging, and of the International Council of Nurses, in the campaign “*Nurses-the leading voice: health as a human right*”, for having launched the challenges to affirm people’s human rights, including the rights of the elderly^(^ 43 ^)^, especially in the process of life and death.

The results of this study can contribute to direct and stimulate health professionals, especially nursing, in care for the elderly who contracted the novel disease, stimulating a dialogue based on demystifying the disease and ensuring an approach focused on the elderly and family members. Through internet access, the elderly have viewed content that can generate misinformation, and health professionals, especially nurses, should be trained to take care of this vulnerable population.

One limitation of this study is the absence of epidemiological studies to identify how the virus spread in each of the countries investigated, which makes it difficult to understand the behavior of the disease in general, and particularly in the elderly population. Another limitation is the charge of high access fees by some of the most well-known newspapers in France, Italy, and the United States, making it impossible to collect newspaper articles on these sites.

## Conclusion

The newspaper articles on hospital care for the elderly with COVID-19 were quickly disseminated in all countries and point to the need to reorganize the health systems for care to the elderly population due to their frailty and the lack of qualified professionals to offer care to this clientele.

The new coronavirus pandemic, COVID-19, reached all countries gradually and the journalistic materials analyzed disclosed the worrying reality of health care for the elderly population and the lack of training of health professionals in this situation. The number of deaths increased gradually and brought health systems closer to collapse day after day, especially in Europe, where the proportion of elderly people is higher. This situation imposed an ethical dilemma on health professionals which these communication vehicles also disseminated widely: deciding between the life and death of the elderly.

The proposal of the theme was the elderly, but the nurse was only mentioned in the newspapers of two countries, the United States and Portugal, although in a restricted manner. The pandemic in the 21^st^ century brings us important reflections for the planning of health systems, the preparation and valuation of professionals for care to people of different age groups, especially the elderly. Thus, one of the challenges of society, health managers and health professionals is the implementation of policies appropriate to the elderly, which ensure their rights. At the same time, and no less important, health professionals need to be guaranteed the right and the duty to follow the ethical precepts of human rights, according to the oath of the profession, so as not to harm the principles of human dignity.

## References

[1] United Nations Medical Directors (2020). Novel Coronavirus (2019-nCoV) prevention Recommendations for UN
Personnel, Families and Visitors.

[2] World Health Organization (2020). Prevención y control de infecciones en los centros de atención de larga
estancia en el contexto de la COVID-19.

[3] Wu Z, McGoogan JM (2020). Characteristics of and important lessons from the coronavirus
disease 2019 (COVID-19) outbreak in China: summary of a report of 72 314
cases from the Chinese Center for Disease Control and
Prevention. JAMA.

[4] World Health Organization (2020). Report of the WHO-China joint mission on Coronavirus disease 2019
(COVID-19).

[5] Centers for Disease Control and Prevention (2020). Interim Clinical Guidance for Management of patients with confirmed
coronavirus diseases (COVID-19).

[6] MacLaren G, Fisher D, Brodie D (2020). Preparing for the most critically ill patients with COVID-19: The
potential role of extracorporeal membrane oxygenation. JAMA.

[7] Organización Mundial de la Salud (2020). Covid-19: Cronología de la actuación de la OMS.

[8] Johns Hopkins University & Medicine (2020). Coronavirus resource center.

[9] Organização Pan-Americana da Saúde (2020). Folha informativa - COVID-19 (doença causada pelo novo
coronavírus).

[10] Vergano M, Bertolini G, Gianini A, Gristina GR, Livigni S, Mistraletti G (2020). Clinical Ethics recommendations for the allocation on intensive
care treatments in exceptional, resource-limited circumstances: the Italian
perspective during the COVID-19 epidemic. Crit Care.

[11] Miller FG (2020). Why I Support Age-Related Rationing of Ventilators for Covid-19
Patients.

[12] Jaramillo ACP (2015). La prensa escrita y la comunicación en salud. Hacia Promoc Salud.

[13] Tong A, Sainsbury P, Craig J (2007). Consolidated criteria for reporting qualitative research (COREQ):
a 32-item checklist for interviews and focus groups. Int J Qual Health Care.

[14] Braun V, Clarke V (2006). Using thematic analysis in psychology. Qual Res Psychol..

[15] Ratinaud P (2020). IRaMuTeQ: Interface de R pour les analyses multidimensionnelles de
textes et de questionnaires.

[16] Reinert M (1990). Alceste, une méthodologie d’analyse des données textuelles et une
application: Aurelia de Gerard de Nerval. Bull Methodol Sociol.

[17] Ministério da Saúde (BR) (2016). Resolução n° 510, de 7 de abril de 2016.

[18] World Health Organization (2013). A universal truth: no health without a workforce.

[19] Lloyd SP, Ebrahim S, Geffen L, McKee M (2020). Bearing the brunt of covid-19: older people in low and middle
income countries. BMJ.

[20] Freitas ARR, Napimoga M, Donalisio MR (2020). Assessing the severity of Covid-19. Epidemiol Serv Saúde.

[21] Emanuel EJ, Persad G, Upshur R, Thome B, Parker M, Glickman A (2020). Fair allocation of scare medical resources in the time of
Covid-19. N Engl J Med.

[22] EuroStat (2020). A look at the lives of the elderly in the EU today.

[23] Ministerio de Sanidad, Consumo y Bienestar Social (ES) (2020). Situación actual coronavirus. Actualización n. 76. Enfermedad por el
coronavirus (COVID-19).

[24] Medicins Sans Frontières (2020). Spain must urgently improve the care of elderly in COVID-19
response.

[25] Santé Publique France (2020). COVID-19: point épidémiologique du 15 mars 2020.

[26] Etard JF, Vanhems P, Atlani-Duault L, Ecochard R (2020). Potential lethal outbreak of coronavirus disease (COVID-19) among
the elderly in retirement homes and long-term facilities, France, March
2020. Eurosurveillance.

[27] Campos LP, Lins T (2020). Portuguese Pandemic: an account of Covid-19 in
Portugal. Espaço Economia.

[28] Organização das Nações Unidas (2020). OMS realça quarentena para o bem de afetados por COVID-19 em
Moçambique.

[29] Centers for Disease Control and Prevention (2020). Cases in the U.S..

[30] Michigan Data (2020). Coronavirus.

[31] Illinois Department of Public Health (2020). Coronavirus disease 2019 (Covid-19).

[32] Centers for Disease Control and Prevention (2020). Morbidity and Mortality Weekly Report: severe outcomes among patients
with Coronavirus disease 2019 (COVID-19) - United States, February 12 -
March 16, 2020.

[33] Ministério da Saúde (BR) (2020). Painel Coronavírus.

[34] Barros L, Rivetti LA, Furlanetto BH, Teixeira EM, Welikow A (2020). COVID-19: General guidelines for cardiovascular surgeons
(standard guidelines - subject to change). Braz J Cardiovasc Surg.

[35] Oliveira WK, Duarte E, França GVA, Garcia LP (2020). How Brazil can hold back COVID-19. Epidemiol Serv Saúde.

[36] Dooley B, Rich M, Inoue M (2020). In graying Japan, many are vulnerable but few are being tested.

[37] Anderson RM, Heesterbeek H, Klinkenberg D, Hollingsworth TD (2020). How will country-based mitigation measures influence the course
of the COVID-19 epidemic?. Lancet.

[38] World Health Organization (2020). Considerations for quarantine of individuals in the context of
containment for coronavirus disease (COVID-19).

[39] World Health Organization (2020). Statement on the second meeting of the International Health Regulations
(2005) Emergency Committee regarding the outbreak of novel coronavirus
(2019-nCoV).

[40] Farias HSF (2020). The advancement of Covid-19 and social isolation as a strategy to
reduce vulnerability. Espaço Economia.

[41] Garcia LP (2020). Use of facemasks to limit COVID-19 transmission. Epidemiol Serv Saúde.

[42] Kucharski AJ, Russel TW, Diamond C, Liu Y, Edmunds J, Funk S (2020). Early dynamics of transmission and control of COVID-19: a
mathematical modelling study. Lancet Infect Dis..

[43] Rodrigues RAP (2019). Healthy aging and the exercise of human rights. Ver. Latino-Am. Enfermagem.

